# Exposure to di-2-ethylhexyl phthalate (DEHP) increases the risk of cancer

**DOI:** 10.1186/s12889-024-17801-w

**Published:** 2024-02-10

**Authors:** Luchen Yang, Xiaoyang Liu, Zhufeng Peng, Zhenghuan Liu, Pan Song, Jing Zhou, Kai Ma, Yunfei Yu, Qiang Dong

**Affiliations:** Department of Urology, Institute of Urology, West China Hospital of Sichuan University, No. 37, Guoxue Lane, Wuhou District, Chengdu, 610041 China

**Keywords:** Cancer, Di-(2-ethylhexyl) phthalate (DEHP), Metabolites, NHANES, Epidemiology

## Abstract

Cancer is a major socioeconomic burden that seriously affects the life and spirit of patients. However, little is known about the role of environmental toxicant exposure in diseases, especially ubiquitous di-(2-ethylhexyl) phthalate (DEHP) which is one of the most widely used plasticizers. Hence, the objective of this study was to assess the potential association between cancer and DEHP. The data were collected using the 2011–2018 National Health and Nutrition Examination Survey (NHANES) data (*n* = 6147), and multiple logistic regression was conducted to evaluate the association. The concentrations of DEHP were calculated by each metabolite and split into quartiles for analysis. After adjusting for confounding factors, DEHP was significantly associated with an increased risk of cancer prevalence, and the metabolites of DEHP showed similar results (OR > 1.0, *p* < 0.05). Simultaneously, the association remained when the analyses were stratified by age and sex, and the risk of cancer appeared to be higher in male patients. In addition, further analysis suggested that DEHP exposure obviously increased the risk of female reproductive system cancer, male reproductive system cancer, and other cancers (OR > 1.0, *p* < 0.05) but not skin and soft tissue cancer. DEHP exposure is associated with the risk of cancer, especially female reproductive system cancer, male reproductive system cancer and other cancers.

## Introduction

As a major public health problem worldwide and the second leading cause of death in the United States, cancer threatens the lives of millions of people and causes a serious social and economic burden. Approximately 1,898,160 cancer cases were diagnosed in 2021 according to the statistics of the United States, of which 608,570 cases died. Simultaneously, the statistics predict that there will be more new cases and deaths [1]. Despite the diversity of cancers, many epidemiological factors have been identified to be associated with cancer, and the incidence of some types of cancer decreased significantly by intervening in these factors. The number of male patients with cancer decreased from the 1990s until approximately 2013, and the cancer incidence remained stable. Simultaneously, the overall cancer incidence in women has slightly increased in recent years after remaining stable over the past few decades [2]. The slow or sustained growth of the overall incidence rate reflects the control of some cancers. Compared with women, lung cancer incidence declines twice as fast in men by controlling tobacco exposure [3]. A previous study [4] suggested that 71% liver cancer can be potentially preventable by decreasing risk factor exposure, such as hepatitis B virus, hepatitis C virus, cigarette smoking and excess alcohol consumption. Tobacco smoking and occupational or environmental exposure to certain chemicals significantly increased bladder cancer incidence [5]. At present, increasing concerns have been raised about cancer-related risk factors, especially in environmental and occupational exposure, because of their potential roles in disease prevention. Because of the environmental pollution deterioration caused by industry, researchers observed excessive emissions of air pollutants in local factories and calculated an obvious health risk by using AERMOD modeling, and they conducted a study suggested that exposure to ambient air pollution obviously increased the thyroid cancer incidence in women in local [6–8]. In addition, another study including different ambient air pollutants found a significantly association between air pollutant exposure and the risk of ovarian cancer [9].

As one of the most widely used plasticizers, di-2-ethylhexyl phthalate (DEHP) is an important phthalate that can improve the pliability, flexibility, and elasticity of plastics [10]. DEHP exposure is ubiquitous either in plastic production or the environment, and this phthalate can be detected not only in household products, medical devices, rubbing alcohol, liquid detergents, and food packaging but also in food, air pollutants, industrial sewage, soil, and rivers [11]. Hence, the human population is continuously exposed to the phthalate. DEHP first increases wide concerns because of its endocrine disrupting properties, which potentially cause a series of disorders in multiple organs, including the thyroid, testis, uterus, ovary, liver, and nerve, and a daily intake of 50 μg/kg of body weight/day for DEHP may result in adverse effects on human health [12, 13]. This toxicant enters the human body mainly through ingestion, inhalation, and dermal exposure and is metabolized into four main substances, Mono-2-ethylhexyl phthalate (MEHP), Mono-(2-ethyl-5-carboxypentyl) phthalate (MECPP), Mono-(2-ethyl-5-hydroxyhexyl), and Mono-(2-ethyl-5-oxohexyl) phthalate (MEOHP), and eventually plays toxic role [14].

DEHP exposure has been reported to be associated with many diseases in vivo and in vitro experiments, especially in cancer [15]. The carcinogenic effects induced by long-term exposure to DEHP have been observed in rodents [16]. Cristina Voss et al*.* [17] found that lifelong (159 weeks) exposure to DEHP induced liver and testicular tumors in male SD rats, and the multiplicity of tumors increased with time. Simultaneously, previous studies [18, 19] suggested that DEHP can promote prostate cancer cell proliferation in vitro, and MEHP, the major metabolite of DEHP, could advance the progression of prostate cancer in which the effects increased with prolonged exposure time. In vivo, Bin Xia et al*.*[20] indicated that the susceptibility of prostate carcinogenesis increased in male SD rat offspring to exposure to DEHP in utero and lactation. Moreover, similar results have also been observed in breast cancer. Previous studies [21, 22] found that DEHP and its metabolite increased the proliferation of epithelial breast cancer cells without inducing apoptosis, and coexposure to DEHP and bisphenol A, another common plasticizer, increased the risk and reduced the latency of mammary tumors in female rats.

However, the association of DEHP exposure and overall cancer is unknown, and little epidemiological evidence is available to support the carcinogenic effects on humans. Hence, we used a nationally representative sample from the 2011–2018 National Health and Nutrition Examination Survey (NHANES) data to assess the role of DEHP, the most widely studied EDC, in overall and different systematic cancer prevalence.

## Materials and methods

### Study population

We estimated the association between urinary DEHP metabolites and cancer by analyzing four circles (2011–2012, 2013–2014, 2015–2016 and 2017–2018) of NHANES, in which the data represent the health and nutritional status of the civilian, noninstitutionalized U.S. population. The NHANES program was initiated by the CDC’s National Center for Health Statistics and is a national, cross-sectional survey that designs a series of laboratory tests, physical examinations and questionnaires to collect information that is sampling-probability based. All participants were asked to sign informed consent forms, and data are publicly accessible on the NHANES website (https://www.cdc.gov/nchs/nhanes/index.htm). NHANES was approved by the NCHS Research Ethics Review Board (ERB).

**Fig. 1:**
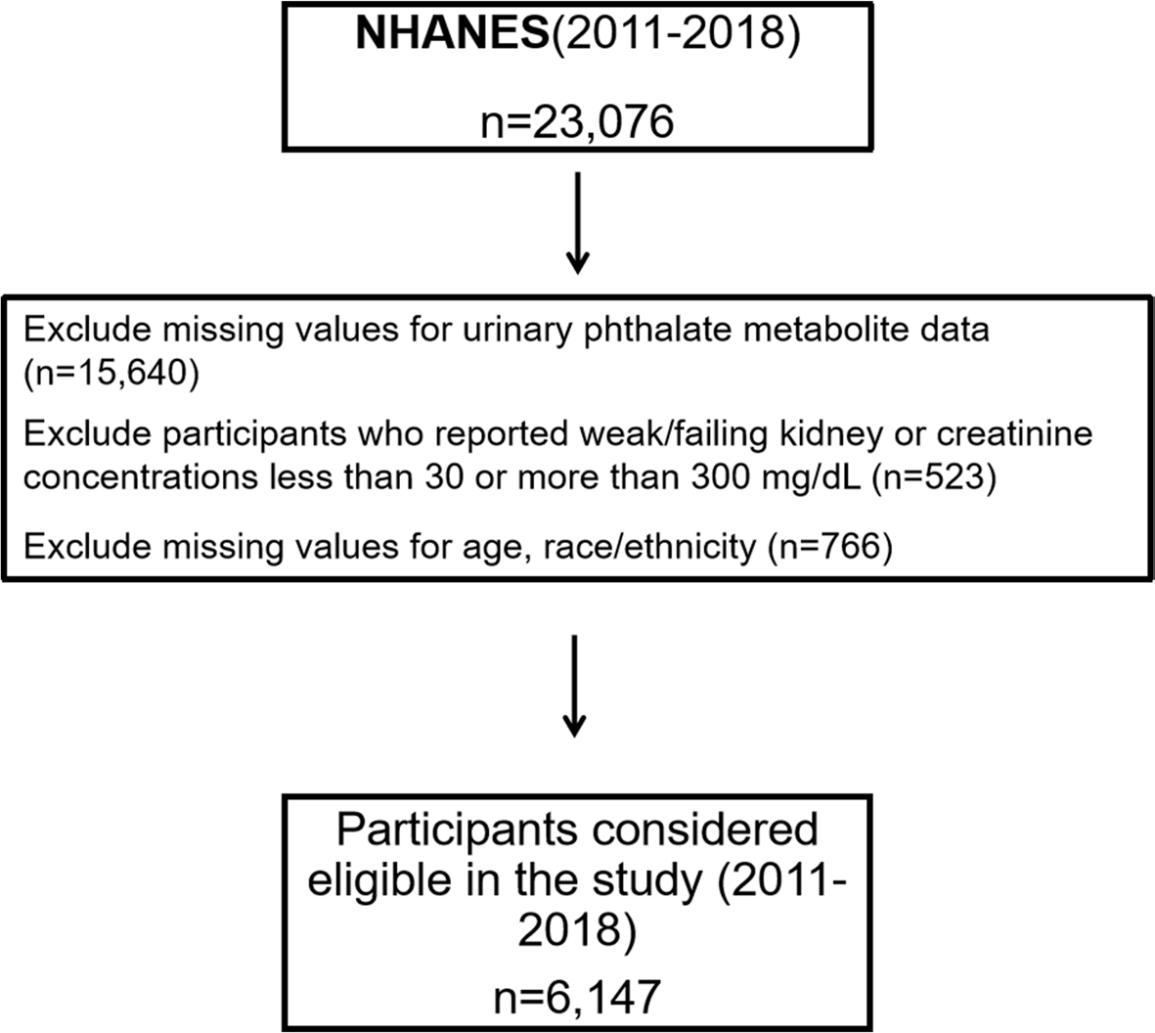
Flow chart of individuals included in our final analysis, NHANES 2011–2018

In our study, participants who completed the “Medical Conditions- Ever told you had cancer or malignancy” questionnaire were considered. Of the 23,076 participants whose related data were available, further exclusion criteria were set up: 1. urinary phthalate metabolite data lost (*n* = 15,640); 2. participants who reported weak/failing kidney or creatinine concentrations less than 30 or more than 300 mg/dL(*n* = 523); and 3. missing information on age, race/ethnicity (*n* = 766). The exclusion criteria eventually resulted in 6147 participants (Fig. 1). The levels of each metabolite were creatinine-corrected to avoid the potential creatinine-related biases resulting from measuring phthalate exposures by assessing urinary metabolite levels [23]. Table 1 details the study population characteristics by cancer status.

**Table 1:** Study population characteristics by cancer status; NHANES 2011–2018. Numbers that do not add up to 100% are attributed to missing data

	**Cancer status**					***P*-value**
	No	Yes	(%)	Total	(%)	
**Population**	5578	569	9.3%	6147	100%	
**Gender**						< 0.001
Male	2650	276	9.4%	2926	47.6%	
Female	2928	293	9.1%	3221	52.4%	
**Urine Creatinine (mg/dl)**	117.9 ± 69.6	105.5 ± 61.3				0.011
**AGE (years)**						< 0.001
20–35	1540	17	1.1%	1557	25.3%	
36–50	1473	50	3.3%	1523	24.8%	
51–64	1400	138	9.0%	1538	25.0%	
64–80	1165	364	23.8%	1529	24.9%	
**BMI (kg/m2)**						< 0.001
≥ 30.0	2142	225	9.5%	2367	38.5%	
25.0–29.9	1846	186	9.2%	2032	33.1%	
18.5–24.9	1484	145	8.9%	1629	26.5%	
< 18.5	106	13	10.9%	119	1.9%	
**Race/ethnicity**						< 0.001
Mexican American	786	38	4.6%	824	13.4%	
Other Hispanic	586	46	7.3%	632	10.3%	
Non-Hispanic White	2008	362	15.3%	2370	38.6%	
Non-Hispanic Black	1227	81	6.2%	1308	21.3%	
Other Race	971	42	4.1%	1013	16.5%	
**Education**						0.094
Less than high school	1266	97	7.1%	1363	22.2%	
High School Grad/GED or Equivalent	1210	133	9.9%	1343	21.8%	
More than high school	3096	337	9.8%	3433	55.8%	
**Marital Status**						< 0.001
Married	2850	322	10.2%	3172	51.6%	
Single	2248	226	9.1%	2474	40.2%	
Living with a partner	480	20	4.0%	500	8.1%	
**Poverty ratio**						< 0.001
< 1.0	1099	80	6.8%	1179	19.2%	
≥ 1.1	3921	435	10.0%	4356	70.9%	
**Hypertension Status**						< 0.001
Yes	1930	333	14.7%	2263	36.8%	
No	3642	234	6.0%	3876	63.1%	
**Diabetes Status**						< 0.001
Yes	703	127	15.3%	830	13.5%	
No	4725	426	8.3%	5151	83.8%	
**Coronary Heart Disease Status**						< 0.001
Yes	184	51	21.7%	235	3.8%	
No	5383	516	8.7%	5899	96.0%	
**Smoke at Least 100 Cigarettes in Life**						< 0.001
Yes	2254	306	12.0%	2560	41.6%	
No	3319	263	7.3%	3582	58.3%	
**Alcohol**						< 0.001
Yes	39	1	2.5%	40	0.7%	
No	5500	548	9.1%	6048	98.4%	
**∑DEHP Quartiles**						0.015
Lowest Quartile	1428	104	6.8%	1532	24.9%	
Second Quartile	1400	142	9.2%	1542	25.1%	
Third Quartile	1383	167	10.8%	1550	25.2%	
Highest Quartile	1367	156	10.2%	1523	24.8%	

### DEHP metabolite measurements

Phthalate metabolite measurements were randomly conducted in one-third of the participants in NHANES. In our study, four major metabolites of DEHP were evaluated, including MEHP, MECPP, MEOHP, and MEHHP. These four monoester metabolites have been proven to be sensitive and representative biomarkers reflecting DEHP exposure [24]. Considering bias resulting from concentrations below the limit of detection (LOD), we only collected DEHP metabolite data that were detected in at least 75% of the samples. The quantitative detection of all metabolites in urine was performed by high-performance liquid chromatography-electrospray ionization-tandem mass spectrometry (HPLC–ESI–MS/MS), and details are described in the Description of Laboratory Methodology in NHANES. Simultaneously, we corrected urine dilution in our study using creatinine-corrected metabolite concentrations by dividing the DEHP metabolite concentration by the urinary creatinine concentration and consequently, our final unit was ng/mg crt [25].

### Measurement of cancer

Participants were considered to be diagnosed with cancer status when responding “yes” to the question “Ever told you had cancer or malignancy”, and the data on the kinds and frequency of cancer were also collected. In addition, we excluded those who did not know the answer, refused to answer the question, or had a missing value.

### Statistical analysis

We adapted the statistical packages R (The R Foundation; http://r-project.org; version 3.4.3) and EmpowerStats (www.empowerstats.com; X&Y solution inc) for data analysis. The complex sampling design and weights recommended by NHANES were used, and *P* ≤ 0.05 was considered statistically significant. We calculated weighted frequencies and descriptive statistics and analyzed the concentration of ∑DEHP by dividing the molecular weight of each metabolite and summing and then multiplying by the molecular weight of DEHP:$\left\{{\lbrack\mathrm{MEHP}\times(1/278.34)\rbrack+\lbrack\mathrm{MEHHP}\times(1/294.34)\rbrack+\lbrack\mathrm{MEOHP}\times(1/292.33)\rbrack+\lbrack\mathrm{MECPP}\times(1/308.33)\rbrack}\ast390.56\right\}$ [26]. Natural log transformation of the ∑DEHP and metabolites were used for each analyte prior to analysis because of the strongly right-skewed distribution. Based on the weighted distributions in population study, the quartiles of ∑DEHP and metabolites were computed, and simultaneously, multivariable logistic regression analysis was used. The association of urinary ∑DEHP and cancer was assessed by calculating odds ratios (ORs) and 95% confidence intervals (CIs). Previous study [27] suggested that the cancer prevalence was influenced by various factors including personal characteristics, comorbidities and lifestyle-associated factors, and, hence, we identified general confounding factors, and, all candidate factors were further selected by changing the estimates of each metabolite exposure on cancer by more than 10% in the final model. Three models were constructed: Model 1was the crude model that adjusted for no variable; Model 2 adjusted for socio-demographic factors (gender, age, race/ethnicity, poverty ratio, education, marital status); Model 3 adjusted for variables in Model 2 and BMI, hypertension status, diabetes status, coronary heart disease status, drinking situation and smoke condition. Simultaneously, we further conducted stratified analyses for age and sex and classified different cancers based on the human system. Three kinds of systematic cancers with the largest number of prevalences in our study were selected, and the rest were classified as others. The association between urinary ∑DEHP and these four kinds of cancers was also analyzed.

## Results

The weighted distributions of the study population (*n* = 6147) characteristics of the total sample are detailed in Table 1. Of those 6147 participants, 569 people were diagnosed with cancer, and 5578 people have no history of cancer. The prevalence of cancer was different in different age groups, showing an upward trend with the increase of age, and in the 20–35 age group, the prevalence of cancer was 1.1% and in the 64–80 age group was 23.8%. Simultaneously, in all the populations, non-Hispanic White participants accounted for the majority, which was 38.6%, and also contributed to the highest cancer prevalence (15.3%). In addition, 38.5% of participants who reported that their BMI was over 30 kg/m2, and in all participants, the majority of the poverty ratio was 1.1–5.0. 51.6% of the participants were married, in which the prevalence of cancer was 10.2%. Of those who reported cancer status, 333 reported hypertension, 127 reported diabetes, and 51 reported coronary heart disease, and cancer prevalence was 14.7%, 15.3%, and 21.7%, respectively. Among the participants who smoked and drank, the prevalence was 12% and 2.5% respectively. Table 2 detailed study population distribution by cancer status based on the quartiles of ∑DEHP and metabolites, and the trend of cancer prevalence almost climbed with the increase of the quartiles.

**Table 2:** Study population distribution by cancer status based on the quartiles of ∑DEHP and metabolites; NHANES 2011–2018

		**Cancer status**						
	No *n* = 5578			Yes *n* = 569	(%)	Total	(%)	*P*
∑DEHP								< 0.001
Q1: < 5.1 ng/mg crt	1417			103	6.8%	1520	24.7%	
Q2: 5.1–5.6 ng/mg crt	1397			141	9.2%	1538	25.0%	
Q3: 5.7–6.1 ng/mg crt	1380			166	10.7%	1546	25.2%	
Q4: > 6.1 ng/mg crt	1384			159	10.3%	1543	25.1%	
p trend								
MEHHP								< 0.001
Q1: < 3.6 ng/mg crt	1431			96	6.3%	1527	24.8%	
Q2: 3.6–4.1 ng/mg crt	1362			160	10.5%	1522	24.8%	
Q3: 4.2–4.6 ng/mg crt	1371			149	9.8%	1520	24.7%	
Q4: > 4.6 ng/mg crt	1414			164	10.4%	1578	25.7%	
p trend								
MEHP								0.222
Q1: < 1.9 ng/mg crt	1377			142	9.3%	1519	24.7%	
Q2: 2.0–2.5 ng/mg crt	1393			156	10.1%	1549	25.2%	
Q3: 2.6–3.2 ng/mg crt	1392			148	9.6%	1540	25.1%	
Q4: > 3.2 ng/mg crt	1416			123	8.0%	1539	25.0%	
p trend								
MEOHP								< 0.001
Q1: < 3.1 ng/mg crt	1414			100	6.6%	1514	24.6%	
Q2: 3.1–3.6 ng/mg crt	1405			146	9.4%	1551	25.2%	
Q3: 3.7–4.2 ng/mg crt	1386			158	10.2%	1544	25.1%	
Q4: > 4.2 ng/mg crt	1373			165	10.7%	1538	25.0%	
p trend								
MECCP								0.002
Q1: < 4.1 ng/mg crt	1418			107	7.0%	1525	24.8%	
Q2: 4.1–4.5 ng/mg crt	1386			138	9.1%	1524	24.8%	
Q3: 4.6–5.0 ng/mg crt	1396			157	10.1%	1553	25.3%	
Q4: > 5.0 ng/mg crt	1378			167	10.8%	1545	25.1%	

As shown in Table 3, the association between DEHP exposure and cancer was assessed. Our Model 1 indicated a significant association between ∑DEHP and cancer. In comparison to the lowest quartile, the second, third and highest quartiles of ∑DEHP obviously increased 39%, 65% and 56%, respectively. Simultaneously, the results of multivariable linear regression by each metabolite indicated that all metabolites and all quartiles were statistically associated with an increased risk of cancer, with the exception of the highest quartile of MEHP (OR = 0.84, 95% CI [0.78, 0.90]). The results were stable in Model 3, which adjusted for sociodemographic factors, BMI, hypertension status, diabetes status, coronary heart disease status, drinking situation and smoking condition. Compared with the lowest quartile of ∑DEHP, the other three quartiles indicated were significantly associated with cancer (Q2 OR = 1.17, 95% CI [1.08, 1.27]; Q3 OR = 1.22, 95% CI [1.13, 1.33]; Q4 OR = 1.29, 95% CI [1.19, 1.40]), and except for the second and third quartiles of MECCP, all quartiles of different metabolites revealed a significant association with cancer, including the highest quartile of MEHP which increased 14% compared with the lowest quartile.

**Table 3:** Association [OR (95% CI)] between creatinine-corrected DEHP metabolites and cancer; NHANES 2011–2018

	Model 1	Model 2	Model 3
∑DEHP	6147	6147	6147
Q1: < 5.1 ng/mg crt	Reference	Reference	Reference
Q2: 5.1–5.6 ng/mg crt	1.39 (1.29, 1.50)	1.20 (1.10, 1.30)	1.17 (1.08, 1.27)
Q3: 5.7–6.1 ng/mg crt	1.65 (1.53, 1.78)	1.26 (1.16, 1.37)	1.22 (1.13, 1.33)
Q4: > 6.1 ng/mg crt	1.56 (1.45, 1.69)	1.32 (1.22, 1.44)	1.29 (1.19, 1.40)
p trend	< 0.001	< 0.001	< 0.001
MEHHP	6147	6147	6147
Q1: < 3.6 ng/mg crt	Reference	Reference	Reference
Q2: 3.6–4.1 ng/mg crt	1.78 (1.65, 1.93)	1.61 (1.49, 1.75)	1.58 (1.45, 1.72)
Q3: 4.2–4.6 ng/mg crt	1.63 (1.51, 1.77)	1.41 (1.29, 1.53)	1.37 (1.25, 1.49)
Q4: > 4.6 ng/mg crt	1.74 (1.61, 1.88)	1.54 (1.41, 1.67)	1.53 (1.41, 1.67)
p trend	< 0.001	< 0.001	< 0.001
MEHP	6147	6147	6147
Q1: < 1.9 ng/mg crt	Reference	Reference	Reference
Q2: 2.0–2.5 ng/mg crt	1.10 (1.03, 1.19)	1.15 (1.07, 1.24)	1.15 (1.06, 1.24)
Q3: 2.6–3.2 ng/mg crt	1.03 (0.96, 1.10)	1.24 (1.15, 1.34)	1.25 (1.16, 1.35)
Q4: > 3.2 ng/mg crt	0.84 (0.78, 0.90)	1.16 (1.07, 1.26)	1.14 (1.05, 1.24)
p trend	< 0.001	< 0.001	< 0.001
MEOHP	6147	6147	6147
Q1: < 3.1 ng/mg crt	Reference	Reference	Reference
Q2: 3.1–3.6 ng/mg crt	1.48 (1.37, 1.60)	1.28 (1.18, 1.39)	1.27 (1.16, 1.38)
Q3: 3.7–4.2 ng/mg crt	1.62 (1.50, 1.75)	1.23 (1.14, 1.34)	1.21 (1.11, 1.32)
Q4: > 4.2 ng/mg crt	1.70 (1.58, 1.84)	1.40 (1.28, 1.52)	1.37 (1.26, 1.49)
p trend	< 0.001	< 0.001	< 0.001
MECCP	6147	6147	6147
Q1: < 4.1 ng/mg crt	Reference	Reference	Reference
Q2: 4.1–4.5 ng/mg crt	1.30 (1.20, 1.40)	1.06 (0.97, 1.15)	1.03 (0.95, 1.12)
Q3: 4.6–5.0 ng/mg crt	1.49 (1.38, 1.61)	1.09 (1.00, 1.18)	1.06 (0.97, 1.15)
Q4: > 5.0 ng/mg crt	1.58 (1.46, 1.70)	1.23 (1.14, 1.34)	1.22 (1.12, 1.32)
p trend	< 0.001	< 0.001	< 0.001

*Q* Quartile. For each of the metabolites, Q1 is the reference. Model 1 adjusted for no variable, which represented our crude model; Model 2 adjusted for sociodemographic factors (age, sex, race/ethnicity, poverty ratio, education, marital status); Model 3 adjusted for sociodemographic factors plus BMI, hypertension status, diabetes status, coronary heart disease status, drinking situation and smoking condition

**Fig. 2:**
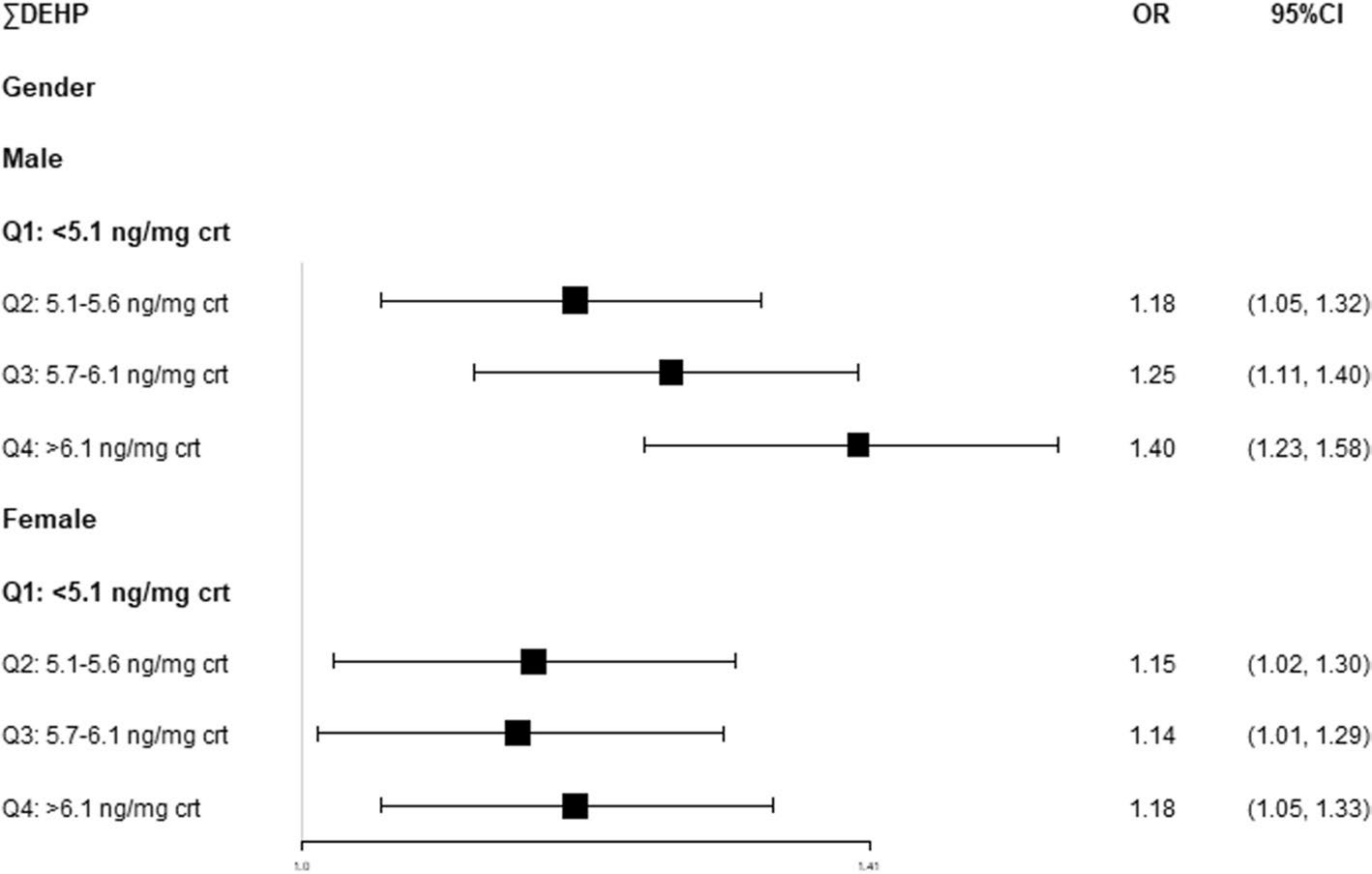
Adjusted ORs and CIs of cancer stratified by gender by concentration of creatinine-corrected DEHP; NHANES 2011–2018

**Fig. 3:**
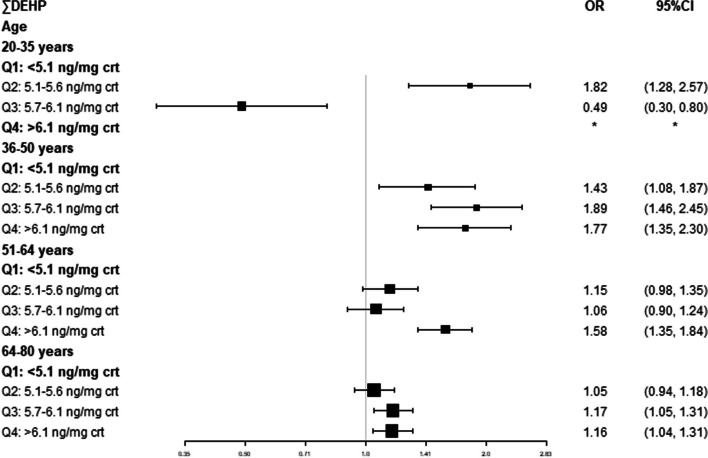
Adjusted ORs and CIs of cancer stratified by age by concentration of creatinine-corrected DEHP; NHANES 2011–2018. * The model failed because of the small sample size

We adjusted for all relative factors and conducted further stratified analyses by gender and age based on the ∑DEHP quartiles. We found that compared with the lowest quartile, all quartiles of ∑DEHP significantly increased the risk of cancer either in male patients or female patients, and the risk appeared to be higher in male patients. The second, third and highest quartiles of ∑DEHP obviously increased 18%, 25% and 40% in male patients and 15%, 14% and 18% (Fig. 2). For participants with different ages, the second, third and highest quartiles of ∑DEHP in the 36–50 age group were significantly increased in comparison to the lowest quartile (Q2 OR = 1.43, 95% CI [1.08, 1.87]; Q3 OR = 1.89, 95% CI [1.46, 2.45]; Q4 OR = 1.77, 95% CI [1.35, 2.30]). In the 51–64 age group, only the highest quartile of ∑DEHP showed an obvious association (OR = 1.58, 95% CI [1.35, 1.84]), while in the 64–80 age group, the third quartile increased 17%, and the highest quartile increased 16%. The results in the 20–35 age group were unstable, which resulted from the relatively small sample size (Fig. 3).

**Fig. 4:**
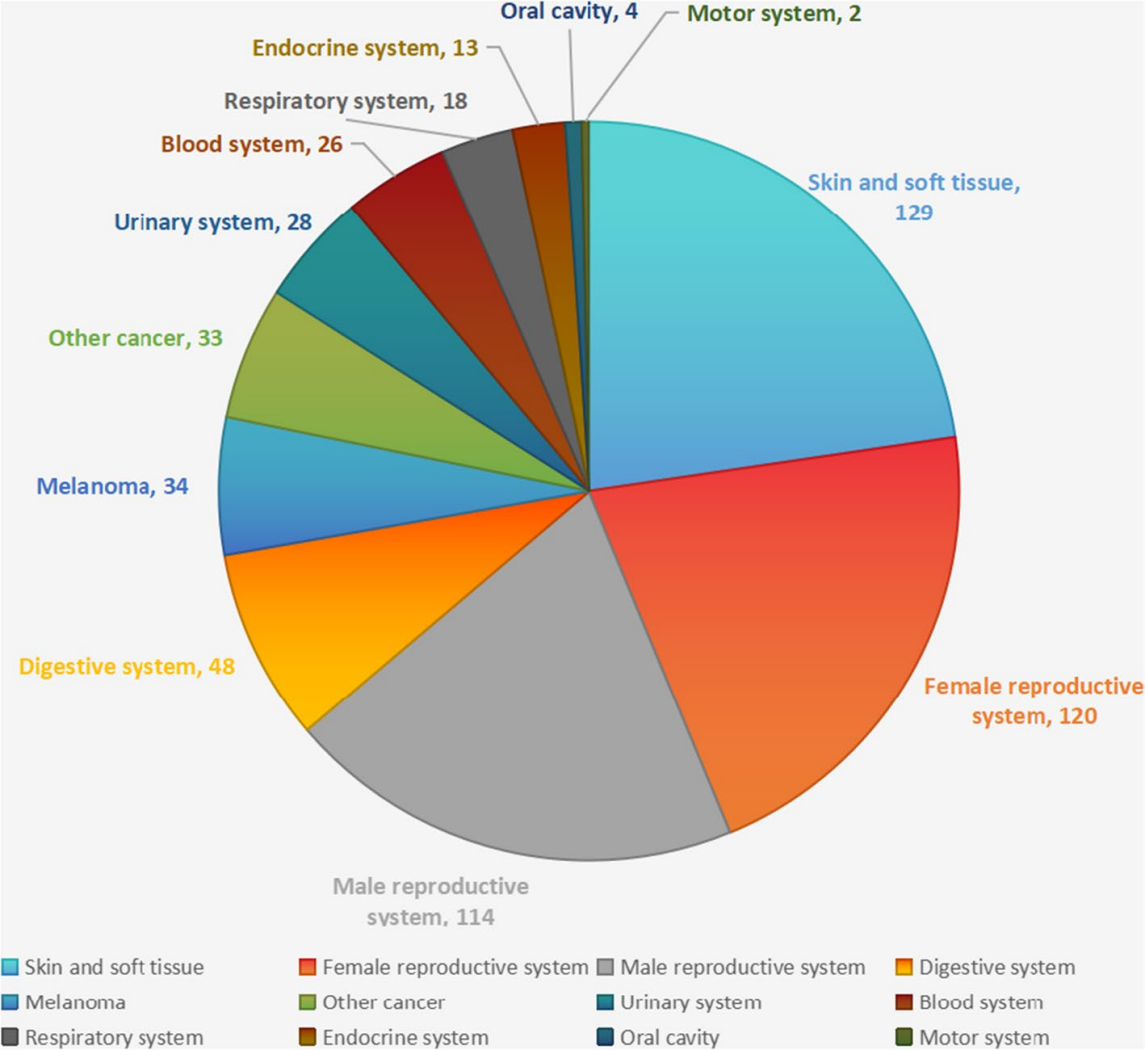
Types of cancer in our study

**Fig. 5:**
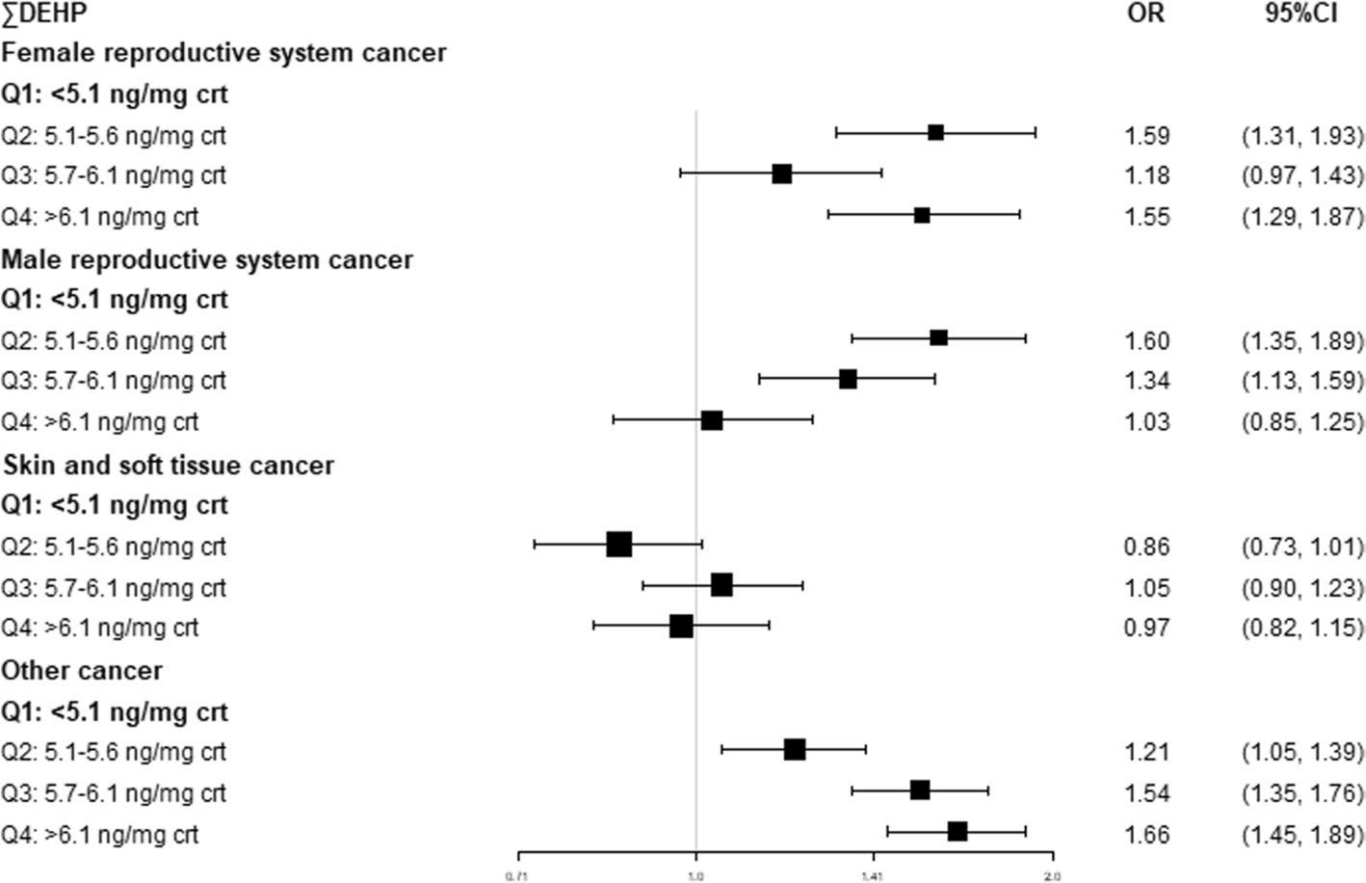
Association [OR (95% CI)] between DEHP and four main types of cancer; NHANES 2011–2018. ORs and CIs were adjusted for all confounding factors

In our study, the three most common cancers were from skin and soft tissue (*n* = 129), the female reproductive system (*n* = 120), and the male reproductive system (*n* = 114), and the remaining cancers were classified as others (Fig. 4). After adjusting for all relative factors, we evaluated the association between these four kinds of cancers and ∑DEHP, and we found that compared with the lowest quartile, the second and highest quartiles of ∑DEHP increased the risk of female reproductive system cancer 59% and 55%, respectively. Simultaneously, the second and third quartiles of ∑DEHP increased the risk of male reproductive system cancer by 60% and 34%, respectively, and a significant association between other cancers and ∑DEHP was also observed. However, no significant association was identified between ∑DEHP and skin and soft tissue cancer (*P* > 0.05) (Fig. 5). Of those 569 participants who were diagnosed with cancer, 509 had only one type of cancer, while the remaining 60 had two or more types, hence, a further study was conducted to assess whether there was an association between high concentration of ∑DEHP and multiple numbers of cancers. However, there was no significant difference in ∑DEHP concentration between patients with only one cancer and patients with two or more cancers (*P* > 0.05) (Table 4).

**Table 4:** Association [OR (95% CI)] between creatinine-corrected DEHP metabolites and the frequency of cancer; NHANES 2011–2018

	Model 1	Model 2	Model 3
∑DEHP	569	569	569
Q1: < 5.1 ng/mg crt	Referent	Referent	Referent
Q2: 5.1–5.6 ng/mg crt	1.46 (1.17, 1.82)	1.24 (0.99, 1.57)	1.25 (0.99, 1.58)
Q3: 5.7–6.1 ng/mg crt	0.94 (0.74, 1.19)	0.76 (0.60, 1.01)	0.74(0.58, 1.00)
Q4: > 6.1 ng/mg crt	1.34 (1.07, 1.68)	1.16 (0.91, 1.47)	1.10 (0.86, 1.41)
p trend	0.384	0.783	0.493

Q, quartile. Q1 is the reference. Model 1 adjusted for no variable, which represented our crude model; Model 2 adjusted for socio-demographic factors (age, gender, race/ethnicity, poverty ratio, education, marital status); Model 3 adjusted for sociodemographic factors plus BMI, hypertension status, diabetes status, coronary heart disease status, drinking situation and smoking condition

## Discussion

We assessed the association between DEHP exposure and cancer using a nationally representative cross-sectional study. Our results indicated an association between DEHP exposure and cancer status, and DEHP can increase the risk of cancer. Simultaneously, the association is significant in different sexes and different age periods, and the risk appears to be higher in male patients. In our study, further analysis suggested that DEHP exposure obviously increased the risk of female reproductive system cancer, male reproductive system cancer and other cancers, except for the skin and soft tissue cancer. In addition, no association was observed between DEHP exposure and the frequency of cancer. Studies on this topic are scarce, and the findings are conflicting due to the complexity of DEHP exposure and cancer.

Cancer is a major socioeconomic burden that seriously affects the life and spirit of patients, and the underlying mechanism is unclear. Simultaneously, widespread concern has been raised about the association between the risk of cancer and environmental toxicant exposure. DEHP is one of the most studied toxicants, and its potential carcinogenicity has been assessed in different cancers, although no epidemiological report on the association between the overall prevalence of cancer and DEHP exposure is available. In a population-based nested case–control study, it was found that DEHP could increase the risk of prostate cancer by analyzing the concentrations of each metabolite of DEHP in urine [28]. Over million woman-years of follow-up, Thomas P Ahern et al*.* [29]*.* found that more than 10,000 cumulative mg of DEHP was associated with a nearly twofold increase in the rate of estrogen receptor-positive breast cancer. Simultaneously, it was found that DEHP exposure fivefold increased the risk of papillary thyroid cancer by evaluating 111 cases, and based on another study, MEHHP, a metabolite of DEHP, was observed to be associated with the risk of urothelial cancer in chronic kidney disease patients [30, 31]. In addition, in vitro and in vivo experiments have also indicated the potential carcinogenicity of DEHP. Mice were continuously exposed to DEHP for 22 months, and the prevalence of liver tumors was significantly higher than that in the control group [32]. Hsin-Pao Chen et al*.* [33] found that the metastasis of colon cancer cells could be enhanced by DEHP and MEHP, and the effects of chemotherapeutic drugs were decreased by these toxicants. These results indicated a risk role of DEHP in the prevalence of cancer, which is consistent with our study.

Our study suggested a positive association between DEHP and the reproductive system and other cancers. The potential mechanisms by which DEHP causes cancer may include activation of nuclear receptors and peroxisome proliferator-activated receptor α, interference with estrogen receptor α and aryl hydrocarbon receptor, and induction of oxidative stress [34]. Breast cancer cells were reported to significantly proliferate in DEHP- and MEHP- treated groups, and the protein levels of isoform A of the progesterone receptor (PR) and nuclear levels of PR in the cells also increased [21]. Simultaneously, DEHP has been reported to not only mediate drug resistance by activating the vinculin/aryl hydrocarbon receptor (AhR)/ERK signaling pathway but also enhance susceptibility to breast cancer by upregulating the Esr1/HDAC6 pathway in female rats [22, 35]. Additionally, an in vitro experiment suggested that peroxisome proliferator-activated receptor-γ (PPAR-γ) could be stimulated by repeated exposure to MEHP and that PPAR-γ plays a vital role in prostate cancer development and progression [28], while another animal study indicated that the risk of liver tumors was higher in PPARα-null mice exposed to DEHP than in wild-type mice, which suggested that DEHP could activate the PPARα pathway [32].

In addition to the above mechanisms, DEHP is also best known as an endocrine disruptor and can disrupt the balance of steroid hormones, which is significantly associated with endocrine-related cancers, such as breast cancer, prostate cancer, testicular cancer and thyroid cancer [36]. Oral exposure to DEHP can induce testicular toxicity in rodent species, which leads to a decrease in testosterone levels [37]. A study on neonatal ovaries from mice exposed to DEHP found that the levels of testosterone, estrone, and E2 were reduced as a result of a decrease in steroidogenic enzyme levels [38]. Simultaneously, thyroid injury has been reported by DEHP exposure, which changed T3 and T4 levels in SD rats [39]. Moreover, DEHP has been reported to induce oxidative stress in multiple organs, and the hypothalamic pituitary adrenal axis (HPA) can be activated by reactive reactive oxygen species (ROS) with the release of cortisol. This hormone affects the anterior pituitary and reduces the secretion of luteinizing hormone (LH) and follicle-stimulating hormone (FSH) through negative feedback between hypothalamic pituitary gonad (HPG) and HPA axis; hence, the secretion of testosterone in Leydig cells decreases. Simultaneously, the decrease in FSH reduces the release of androgen-binding protein to Sertoli cells and further reduces the level of testosterone. ROS can also affect the hypothalamic pituitary thyroid (HPT) axis and therefore reduce the secretion of the thyroid hormones T3 and T4. T3 can reduce the mRNA level of the acute regulatory protein of steroidogenesis in testes and reduce the production of testosterone, and aromatase activity increases with the production of oxidative stress, which leads to an increase in testicular estradiol levels and prevents the secretion of testosterone [40]. The above results may further support our study that DEHP exposure is a risk factor for female reproductive system cancer, male reproductive system cancer, and other cancers. There are few studies on the association between skin and soft tissue cancer and DEHP exposure. One possible explanation may be that DEHP is not transported across the skin, and cannot be metabolized by esterases in the skin, which may decrease DEHP exposure [41]. However, further study is needed.

Our study has the following limitations. First, the causation of cancer cannot be ascertained from this analysis alone resulting from the cross-sectional study design, and the findings were based on associations which lacked of a causal relationship, and we will conduct further study by combining clinical research and mendelian randomization study [42]. Additionally, the manners of the measurement of cancer status compounded the topic, which was self-reported and measured in a historical way, and self-reported data was potential to lead recall bias, indicating future study should be reasonably designed to include patients with clear diagnoses and collect analyzed samples simultaneously. Furthermore, potential confounding factors that were either not involved in the study or unmeasured cannot be identified such as specific dietary habits, occupation and frequency of exposure to plastic products. Moreover, our study did not consider the cumulative effects of DEHP exposure over time, which should be further studied by using methods in vitro and in vivo. Finally, the cancer prevlaence seemed to be higher than the actual situation in our study due to the inclusion of exclusion criteria, although we used data weighting method to ensure the accuracy of the final data. Despite the limitations of our study, there are strengths. Our study included a large sample size and representative participants living in the United States. Simultaneously, all participants and DEHP metabolites were involved, which covered an 8-year period. Moreover, to our knowledge, this is the first population-based study to examine the association of DEHP exposure and overall cancer status.

## Conclusions

The toxic effects of DEHP and its metabolites on the general population should increase widespread concern because of constant exposure. We evaluated the association between DEHP and cancer in various aspects and concluded that DEHP exposure is a risk factor for the prevalence of cancer in the American population. However, further research is needed, not only because of the limitations of our study but also because of the potential that the status of cancer can be changed by controlling DEHP exposure.

## Data Availability

The datasets generated during and analyzed during the current study are available in the NHANES repository (https://www.cdc.gov/nchs/nhanes/index.htm).
